# Emergence of human-porcine reassortment G9P[19] porcine rotavirus A strain in Guangdong Province, China

**DOI:** 10.3389/fvets.2022.1111919

**Published:** 2023-01-09

**Authors:** Shicheng Luo, Xiuqiao Chen, Guangzhi Yan, Shengnan Chen, Jinghua Pan, Mengyi Zeng, Hui Han, Yajing Guo, Haoquan Zhang, Jiaming Li, Meilian Mo, Mingjie Liu, Liangzong Huang

**Affiliations:** ^1^School of Life Science and Engineering, Foshan University, Foshan, Guangdong Province, China; ^2^Guangdong Findergene Biotechnology Co., Ltd., Foshan, Guangdong Province, China

**Keywords:** porcine rotavirus, G9P[19], reassortment, evolutionary characterization, genome

## Abstract

Group A rotaviruses of the family *Reoviridae* is one of the important intestinal pathogens causing diarrhea in piglets and humans. A human-porcine reassortment rotavirus, GDJM1, was identified from outbreak of diarrhea in suckling piglets and it associated with 60.00% (324/540) morbidity and 20.99% (68/324) mortality in Guangdong Province of China in 2022. Thus, to further characterize the evolutionary diversity of GDJM1, all gene segments were analyzed. The genome constellation was G9-P[19]-I5-R1-C1-M1-A8-N1-T1-E1-H1. Nucleotide sequence identity and phylogenetic analyses showed that the VP6, VP7, NSP4 and NSP5 genes of GDJM1 were the most closely related to the respective genes of porcine strains, with the highest homology ranging from 95.65–98.55% identity. The remaining seven genes (VP1-VP4, NSP1-NSP3) were the most closely related to human strains, with the highest homology ranging from 91.83–96.69% similarity. Therefore, it is likely that GDJM1 emerged as the result of genetic reassortment between porcine and human rotaviruses. To our knowledge, this is the first report that a human-porcine reassortment G9P[19] RVA strain has been identified in mainland China, which providing important insights into evolutionary characterization of G9P[19] RVA strain, and reveals that the strain has a potential risk of cross-species transmission.

## Background

Group A rotaviruses of the family *Reoviridae* is a major cause of acute diarrhea in humans and many animals (1). RVA is a double-stranded RNA virus that consists of 11 gene segments encoding capacity of six structural proteins (VP1–VP4, VP6, and VP7) and six nonstructural proteins (NSP1–NSP6) (2). Sequences of the two outer capsid proteins of RVAs, the glycosylated VP7 and the protease-sensitive VP4, are used to classify the viruses into different G and P genotypes, respectively (3). Currently, 42 G genotypes and 58 P genotypes have been reported to the Rotavirus Classification Working Group (RCWG) (https://rega.kuleuven.be/cev/viralmetagenomics/virus-classification/rcwg). Twelve G genotypes (G1–G6, G8–G12, and G26) and 16 P genotypes (P[1] to P[8], P[11], P[13], P[19], P[23], P[26], P[27], P[32], and P[34]) of RVA have been associated with swine infections or diseases, and the G genotypes of G3, G4, G5, G9 and G11 in pigs are usually combined with the P genotypes of P[5], P[6], P[7], P[13], P[28] and P[32] (4, 5). Moreover, G9P[23] and G9P[7] are the prevalent genotypes in pigs in China (1, 6, 7).

RVA strains are potential zoonosis. At least 10 G genotypes (G1–G5, G9–G12, and G26) and seven P genotypes (P[4], P[6], P[8], P[13], P[14], P[19], and P[25]) of porcine origin have been identified in humans (4). Some genotype combinations, such as G9P[19], have been endemic in humans and pigs in a few regions in Asia (8–11). The first whole genomes of G9P[19] was obtained from samples of diarrheic humans in Thailand (8). Subsequently, G9P[19] was reported in Nepal, Vietnam and Taiwan (9–11). Although human G9P[19] strains are thought to originate from pigs through reassortment, G9P[19] strains in pigs have been rarely identified (porcine G9 strains are usually found in combination with P[7], P[13], P[19], and P[23], and rarely with P[19]) (11).

In this study, we identified G9P[19] RVAs in China for the first time from feces specimens collected from piglets with acute diarrhea. We characterized the whole genome of one of the strains and explored its origin and zoonotic significance.

## Materials and methods

### Samples collection and diarrhea-related pathogens detection

An outbreak of acute diarrhea in suckling piglets occurred in February 2022 in a pig farm in Jiangmen city, Guangdong Province, China, where 36 feces samples were collected from randomly selected sick piglets aged 2–7 days. The samples were homogenized separately in the minimal essential medium (MEM), followed by clarification through centrifugation at 12,000 *g* at 4°C for 10 min. The supernatant was subjected to the total RNA/DNA extraction using the FastPure Viral DNA/RNA Mini Kit (Vazyme, China). The extracted total RNA/DNA was detected using a series of assays of quantitative reverse transcription-polymerase chain reaction (RT-qPCR) as described in a previously published method (Supplementary Table S1) (12–14). The positive amplicons of the RT-PCR assay for the detections of RVAs were sequenced to determine the genotypes of VP7 (15).

### Full genome sequencing

The whole genome of the representative strain of RVAs detected in the porcine samples, GDJM1, was sequenced using the Illumina MiSeq high-throughput sequencing platform. The relevant supernatant was directly subjected to the total RNA extraction using the TRIzol reagent (Invitrogen, CA, USA) according to the manufacturer′s instructions. Following the rRNA depletion using the RiBo-Zero Magnetic Gold kit (Epicentra Biotechnologies, Madison, WI), the remaining RNA was subjected to the RNA-seq using the NEBNext Ultra directional RNA library prep kit (NEB, Ipswich, MA) and then sequenced on the Illumina MiSeq platform (GENEWIZ, Guangzhou, China). The resulting raw reads were trimmed using the software Trimmomatic v0.39 (16), and pig genome sequence reads were removed through mapping against Sscrofa 11.1 using the software Bowtie2 v2.4.1 (17). The remaining reads were *de novo* assembled into contigs using the software MEGAHIT v1.2.9 (18). The assembled contigs were aligned against a customized viral nucleotide reference database of GenBank (Taxonomy ID 10239) or the UniProt virus taxonomic database using blastn/blastx for searching viral sequences.

### Sequence alignment and phylogenetic analysis

RVA genotypes were determined according to the guidelines of RCWG using the online genotyping tool RotaC (http://rotac.regatools.be/) and blastn analysis based on the NCBI non-redundant database. Sequence alignments were performed using the online software MAFFT v7 (19). Sequence comparison was conducted using BioAider v1.423 to analyze sequence identity of nucleotide between GDJM1 and other rotaviruses (20). The neighbor-joining (NJ) tree of 11 segments was constructed using the MEGA X software (21). The reliability of the NJ tree was calculated using 1,000 bootstrap replicates.

## Results

### Detection of RVA infection in feces samples

The outbreak of acute diarrhea in suckling piglets caused 60.00% (324/540) morbidity and 20.99% (68/324) mortality in the pig farm. Severe diarrhea with wasting, yellow watery or semi-solid feces, thin intestinal wall, and mesenteric lymphadenopathy were observed in piglets (Supplementary Figure S1).

The common pathogens of piglet diarrhea, such as porcine epidemic diarrhea virus (PEDV), porcine transmissible gastroenteritis virus (TGEV), porcine rotavirus C (PRVC) and porcine deltacoronavirus (PDCoV), were not found in the feces samples, and RVA was identified in 86.11% (31/36) of the piglet samples by the detection of a series of RT-qPCR assays. Sequences of the viral VP7 gene of the RT-PCR amplicons of the 31 positive piglet feces samples shared 98.30–100.00% nucleotide sequence identity among each other and suggested the RVA belonged to G9 genotype.

### Genome sequences of GDJM1

Unbiased high-through sequencing was performed on Illumina MiSeq platform. A total of 70717084 clean reads from viral genome. A total of 3,310 contigs from viral genome were then generated by *de novo* assembly. The nearly full-length genome sequences of GDJM1 were determined by the RNA-Seq sequencing and the sequences of 11 segments of GDJM1 were deposited in GenBank with the accession numbers OP718288–OP718298. Notably, 0.3% (22221/70717084) of the reads mapped to the genome sequences of GDJM1. Sequence alignment, nucleotide sequence identity analysis, and BLAST analysis all showed that GDJM1 was a G9P[19] strain with the genomic constellation of G9-P[19]-I5-R1-C1-M1-A8-N1-T1-E1-H1 (Table 1). GDJM1 shared the same genomic constellation with the previously characterized G9P[19] strain 2-1 detected in pigs in Taiwan, China (11). According to the nucleotide identity of the coding region sequences, GDJM1 was more close to human RVA strains than porcine RVA strains in the genes of VP1-VP4 and NSP1-NSP3, and more close to porcine RVA strains than humans RVA strains in the genes of VP6, VP7, NSP4, and NSP5 (Table 1).

**Table 1:** The highest nucleotide sequence identities between GDJM1 and other known RVA strains.

**Gene**	**Closely related strains**	**nt (%)**	**Genotype**	**Accession no**.
VP7	RVA/Pig-tc/CHN/GXqz-2/2012/G9	95.65	G9	JX498942.1
VP4	RVA/Human-wt/VNM/NT0073/2007/G9P[19]	91.83	P[19]	DQ887060.2
VP6	RVA/Simian-wt/KNA/08979/2015/G5P[X]	97.14	I5	KY053147.1
VP1	RVA/Human-wt/CHN/GX54/2010/G4P[6]	96.13	R1	KF041441.1
VP2	RVA/Human-wt/LKA/R1207/2009/G4P[6]	96.30	C1	LC389886.1
VP3	RVA/Human-wt/VNM/16020_7/VP3	96.45	M1	KX362691.1
NSP1	RVA/Human-wt/VNM/NT0077/2007/G4P[6]	95.63	A8	LC095916.1
NSP2	RVA/Hu-wt/RUS/Novosibirsk/Nov11-N2687/2011/G4P[6]	93.02	N1	KC020033.1
NSP3	RVA/Human-wt/ZMB/UFS-NGS-MRC-DPRU4723/2014/G5P[6]	96.69	T1	MT271033.1
NSP4	RVA/WildBoar-wt/CZE/P828/2015	97.27	E1	MK283698.1
NSP5	RVA/Pig-wt/VNM/12129_48/NSP5	98.55	H1	KX363314.1

### Phylogenetic analysis of GDJM1

**Figure 1:**
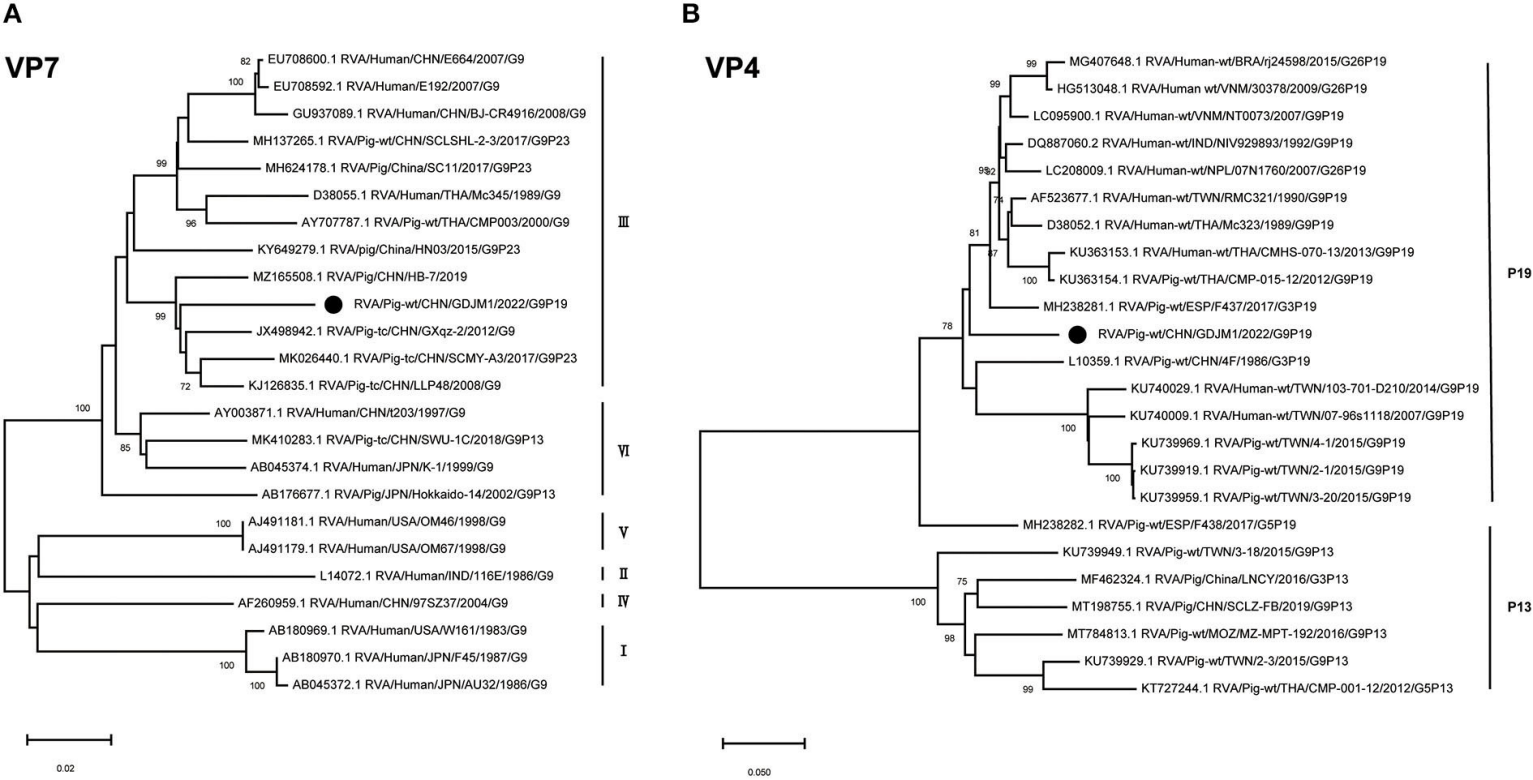
Phylogenic trees based on the nucleotide sequences of VP7 and VP4 genes from GDJM1 identified in this study and those of representative or selected strains of other genotype by MEGA X using the neighbor-joining (NJ) method and 1,000 bootstrap replicates. **(A)** Phylogenic trees based on the nucleotide sequences of the VP7 genes from GDJM1 identified in this study and those of representative strains of other genotype. **(B)** Phylogenic trees based on the nucleotide sequences of the VP4 genes from GDJM1 identified in this study and those of representative strains of other genotype. Bootstrap values above 70% are shown at the branch nodes.

Phylogenetic analysis showed that the VP7 gene of GDJM1 belonged to lineage III within G9 genotype, close to some porcine RVA strains (Figure 1). GDJM1 shared 91.85–95.65% nucleotide sequence identity in the VP7 gene with some porcine lineage III RVA strains within G9 genotype (e.g., sharing 95.65% nucleotide sequence identity with RVA/Pig-tc/CHN/GXqz-2/2012/G9). Phylogenetic analysis revealed that the VP4 gene of GDJM1 were distinct with other G9P[19] strains (Figure 1). GDJM1 shared 85.83–91.83% nucleotide sequence identity with other P[19] RVA strains in GenBank (e.g., sharing 91.83% nucleotide sequence identity with RVA/Human-wt/VNM/NT0073/2007/G9P[19]). GDJM1 shared 86.10–86.21% nucleotide sequence identity in the VP4 gene with some G9P[19] porcine strains (3–20) detected from Taiwan, China. The sequence in GenBank closest to the VP4 gene of GDJM1 was that of RVA/Human-wt/VNM/NT0073/2007/G9P[19], although two strains shared the only 91.83% nucleotide identity in the VP4 gene (Figure 1).

**Figure 2:**
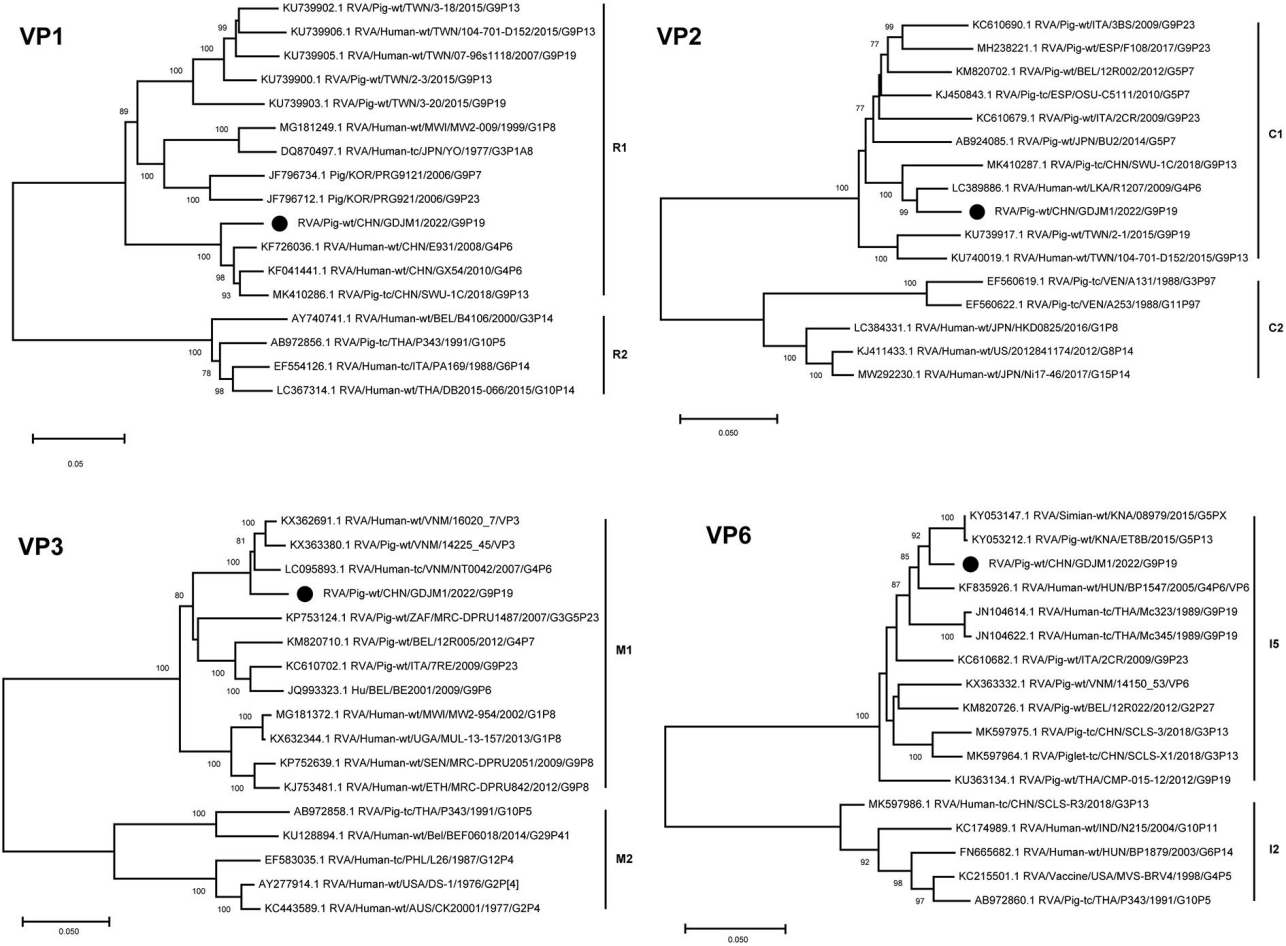
Phylogenic trees based on the nucleotide sequences of VP6 and VP1-VP3 genes from GDJM1 identified in this study and those of representative or selected strains of other genotype by MEGA X using the neighbor-joining (NJ) method and 1,000 bootstrap replicates. Bootstrap values above 70% are shown at the branch nodes.

The VP6 and VP1–VP3 genes of GDJM1 were classified as genotypes I5, R1, C1 and M1, respectively (Figure 2). The VP6 gene of GDJM1 is closely related to the previous porcine RVA strain ET8B and simian RVA strain 08979, with 97.04 and 97.14% nucleotide sequence identities, respectively. The simian strain 08979 might have been derived from interspecies transmission events involving the transmission of ET8B-like RVAs from pigs to simians (22). Therefore, the VP6 segment of GDJM1 are likely to originate from pigs. The VP1 gene of GDJM1 clustered in the same clade, together with the pigs and human strain identified in China. On the contrary, the VP3 gene of GDJM1 clustered in the same clade, together with the pigs and human strain identified in Vietnam. The VP2 segment of GDJM1 was clustered with human strain R1207.

**Figure 3:**
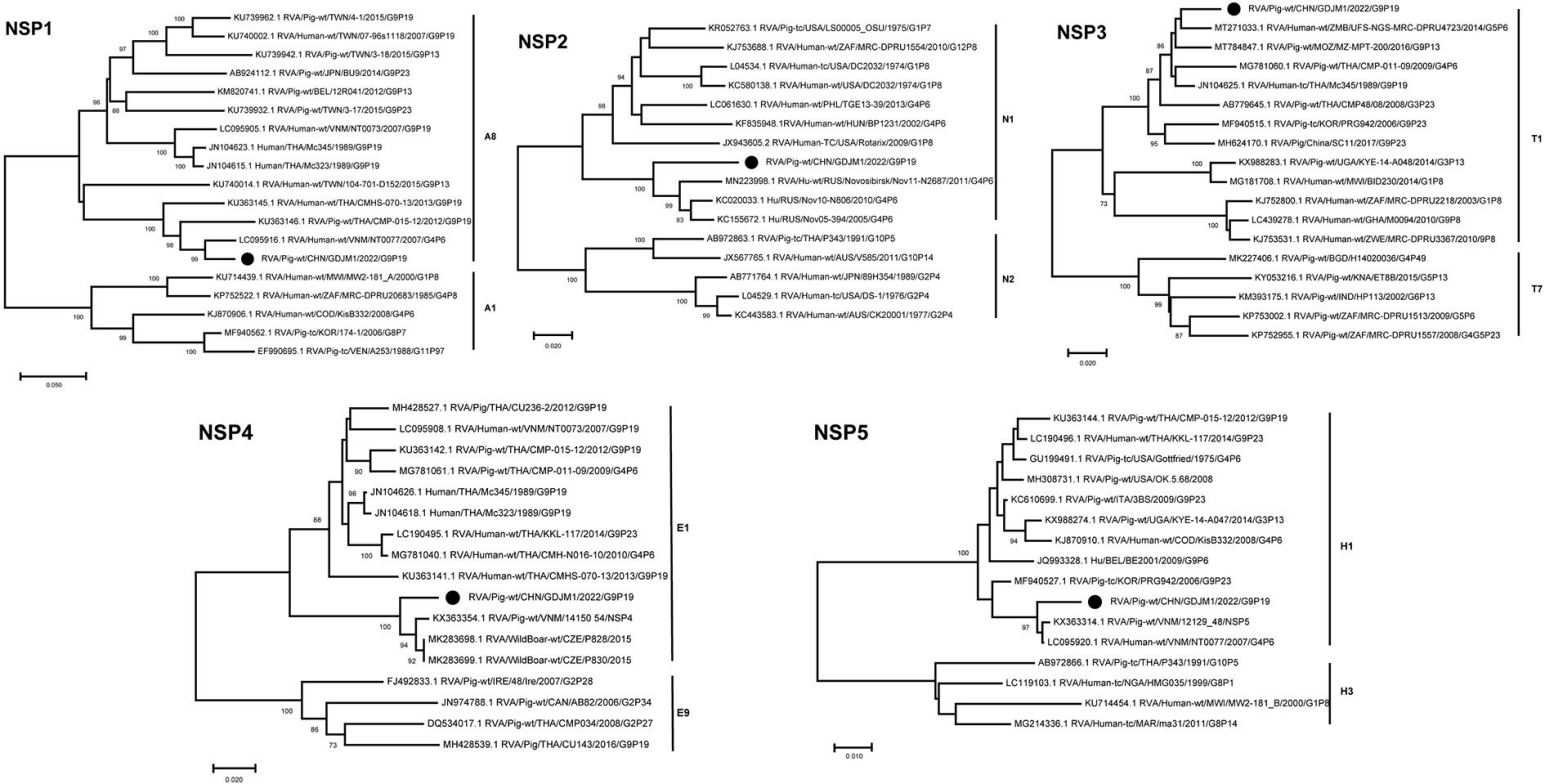
Phylogenic trees based on the nucleotide sequences of NSP1-NSP5 genes from GDJM1 identified in this study and those of representative or selected strains of other genotype by MEGA X using the neighbor-joining (NJ) method and 1,000 bootstrap replicates. Bootstrap values above 70% are shown at the branch nodes.

Phylogenetic analysis showed that the NSP1–NSP5 genes of GDJM1 were clustered into branches of A8, N1, T1, E1 and H1, respectively (Figure 3). The NSP1 segment clustered with the A8 clade, and it is likely that GDJM1 segment has a porcine origin, as porcine isolates dominate this cluster (23). The NSP1 and NSP3 segments of GDJM1 were closely related to the human strains NT0077 and UFS-NGS-MRC-DPRU4723, with nucleotide identities of 95.63% and 96.69%, respectively. The NSP2 segment of GDJM1 formed a unique branch neighboring to human RVA strains (Figure 3). The NSP4 segment was closely related (97.27% nucleotide sequence identity) to porcine strains, and the NSP5 segment was closely related (98.55% and 98.36% nucleotide sequence identity) to the Vietnam porcine strain 12129_48 and the human RVA strain NT0077 (Figure 3).

## Discussion and conclusions

During this outbreak of severe diarrhea in piglets, we found that the pathogen was RVA. Here, amplification of part of the VP7 gene sequence in all porcine RVA-positive samples revealed that the infection originated from the same strain. The whole genome sequence of a G9P[19] RVA strain designated GDJM1 was obtained by metagenomics technology. The genome constellation of GDJM1 was G9-P[19]-I5-R1-C1-M1-A8-N1-T1-E1-H1, which is the same as that observed in previously characterized porcine rotavirus G9P[19] strains in Taiwan, China, but all 11 genes of GDJM1 have relatively low sequence homology with these strains.

There is no evidence of direct transmission of rotavirus between animals and humans, but the reassortment of RVA strains in animals suggests that RVA has potential for human-animal transmission (24–26). Based on homology analysis and phylogenetic analyses of different segment sequences, several segments of GDJM1 were found to be the most closely related to strains derived from humans, although it belonged to the porcine lineage. Further evidence suggests that the porcine RVA strain may be transmissible to humans based on its similarity with several human strains. In addition, the VP4 gene of GDJM1 was independent of the branches of P[19] strains, and was closely related to that of human P[19] that was reported previously in Vietnam (NT0073), but with only 91.83% nucleotide sequence homology between them.

In conclusion, this study is the first to report a porcine RVA G9P[19] strain identified from suckling piglets with severe diarrhea in Guangdong Province of China. Sequence analysis showed that GDJM1 reconstituted the human RVA-like gene fragments resulting in the possibility of cross-species transmission. Due to the lack of RVA G9P[19] strain sequence data derived from humans in Guangdong Province of China, it is difficult to determine whether zoonotic transmission occurs across species in this region. Therefore, G9P [19] strain and its occurrence, pathogenesis and interspecific transmission should be closely monitored.

## Data availability statement

The datasets presented in this study can be found in online repositories. The names of the repository/repositories and accession number(s) can be found in the article/Supplementary material.

## Author contributions

LH designed the project, monitored the project, and wrote and revised the paper. LH, SL, XC, and GY performed all analyses and interpreted the data. XC, GY, HZ, SC, JP, MZ, HH, YG, JL, ML, and MM performed the experiments. LH, SL, and GY collected the samples. All authors revised and approved the paper for publication.
