# Supplementary material for: Emergence of human-porcine reassortment G9P[19] porcine rotavirus A strain in Guangdong Province, China
Source: Front Vet Sci. 2023 Jan 9;9:1111919. doi: 10.3389/fvets.2022.1111919 (PMC9868962; doi:10.3389/fvets.2022.1111919)
Supplement: Supplementary file 1 [file Table_1.docx]

**Supplementary Table S1:** Sequences of primers and probes used in this study.

| Primer name | 5′- sequence −3’ | Target | Reference |
| --- | --- | --- | --- |
| PDCoV-RTF | 5’-CGACCACATGGCTCCAATTC-3’ | M | Zhu JH et al.(2022) |
| PDCoV-RTR | 5’-CAGCTCTTGCCCATGTAGCTT-3’ |  |  |
| PDCoV-RTP | 5’-FAM-CACACCAGTCGTTAAGCATGGCAAGC-BHQ-1-3’ |  |  |
| PEDV-RTF | 5’-GATACTTTGGCCTCTTGTGT-3’ | M | Huang X et al.(2022) |
| PEDV-RTR | 5’-CACAACCGAATGCTATTGACG-3’ |  |  |
| PEDV-RTP | 5’-FAM-TTCAGCATCCTTATGGCTTGCATC-BHQ1-3’ |  |  |
| TGEV-RTF | 5’-TGCCATGAACAAACCAAC-3’ | N |  |
| TGEV-RTR | 5’-GGCACTTTACCATCGAAT-3’ |  |  |
| TGEV-RTP | 5’-FAM-FATAGCACCACGACTACCAAGC-BHQ1-3’ |  |  |
| RVA-RTF | 5’-GCTAGGGAYAAA ATTGTTGAAGGTA-3’ | VP6 | Marthaler D et al.(2014) |
| RVA-RTR | 5’-ATTGGCAAATTT CCTATT CCTCC-3’ |  |  |
| RVA-RTP | 5’-FAM-ATGAATGGA AATAATTTTCAAAC-BHQ1-3’ |  |  |
| RVC-RTF | 5’-ATGTAGCATGATTCACGAATGGG-3’ | VP6 |  |
| RVC-RTR | 5’-ACATTTCATCCTCCTGGGGATC-3’ |  |  |
| RVC-RTP | 5’-FAM-GCGTAGGGGCAAATGCGCATGA-BHQ1-3’ |  |  |
