# Supplementary figures and images for: Emergence of human-porcine reassortment G9P[19] porcine rotavirus A strain in Guangdong Province, China
Source: Front Vet Sci. 2023 Jan 9;9:1111919. doi: 10.3389/fvets.2022.1111919 (PMC9868962; doi:10.3389/fvets.2022.1111919)

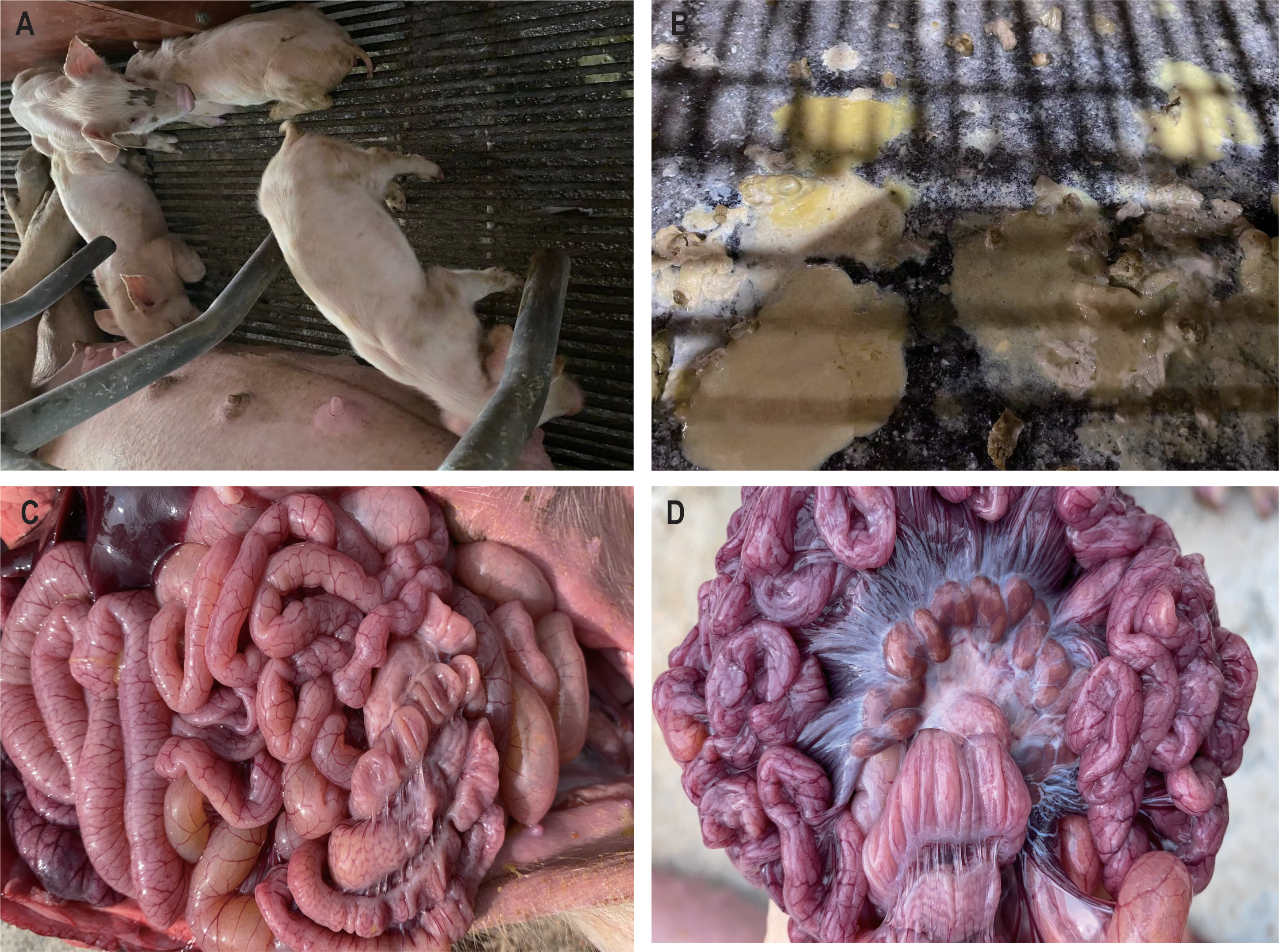

Supplement: Supplementary file 2 [file Image_1.jpg]
